# Direct Comparison of Odor Responses of Homologous Glomeruli in the Medial and Lateral Maps of the Mouse Olfactory Bulb

**DOI:** 10.1523/ENEURO.0449-19.2020

**Published:** 2020-03-04

**Authors:** Tokiharu Sato, Ryota Homma, Shin Nagayama

**Affiliations:** 1 Department of Neurobiology and Anatomy, McGovern Medical School at the University of Texas Health Science Center at Houston, Houston, TX 77030; 2 Department of System Pathology for Neurological Disorders, Brain Research Institute, Niigata University, Niigata 951-8585, Japan

**Keywords:** calcium imaging, glomerulus, map, mouse, olfactory bulb, respiration

## Abstract

Olfactory sensory neurons (OSNs) expressing same-type odorant receptors typically project to a pair of glomeruli in the medial and lateral sides of the olfactory bulbs (OBs) in rodents. This multiple glomerular representation of homologous inputs is considered to have more important functional roles for odor information processing than the redundant backup system. However, a consensus idea is lacking and this hinders interpretation of the phenomenon. In addition, the shared and unique odorant response properties of the homologous glomeruli remain unclear because the majority of medial glomeruli are hidden in the septal OB, and thus it is difficult to directly compare them. OSNs, which express trace amine-associated odorant receptors (TAARs), were recently identified that project to a pair of glomeruli uniquely located in the dorsal OB. In this study, we measured the odorant-induced calcium responses of homologous pairs of TAAR glomeruli simultaneously in anesthetized mice and directly compared their response patterns. We found that they exhibited similar temporal response patterns and could not find differences in onset latency, rise time, decay time, or response amplitude. However, the medial glomeruli had significantly larger respiration-locked calcium fluctuations than the lateral glomeruli. This trend was observed with/without odorant stimulation in postsynaptic neurons of GABAergic, dopaminergic, and mitral/tufted cells, but not in presynaptic olfactory sensory axon terminals. This indicates that, at least in these TAAR glomeruli, the medial rather than the lateral OB map enhances the respiration-locked rhythm and transfers this information to higher brain centers.

## Significance Statement

This study directly compared the odor-evoked responses in paired homologous glomeruli in medial and lateral maps in the mouse dorsal olfactory bulb (OB) using the *in vivo* calcium imaging technique, which revealed that the response amplitude, onset latency, rising time, and decay time were not significantly different between medial and lateral homologous glomeruli. However, postsynaptic but not presynaptic neuronal activity in medial glomeruli had stronger respiratory-locked calcium fluctuations than lateral glomeruli. These differences were also observed in the resting condition without odor stimulation. The results suggest that the medial map neurons enhance respiratory-linked odor representation and transmit it to higher brain centers.

## Introduction

Parallel processing of multiple streams of information improves the speed of processing and provides redundancy for fail-safe operations. Biological parallel streams of information in the brain are not typically identical neuronal circuits but have unique as well as common properties (Kandel et al., 2013). How the brain organizes the distinct processing streams and combines them is not well understood.

Odor information is represented as spatial/temporal glomerular activity patterns on the surfaces of the olfactory bulbs (OBs). In rodents, olfactory sensory neurons (OSNs) expressing the same types of odorant receptors, among ∼1000 repertoires (Buck, 1996), convert chemical signals into electrical signals in a respiratory rhythm and project to approximately two glomeruli in the OB: one on the medioventral side and the other on the dorsolateral side. The axons of these OSN projections traverse the medial/septal and lateral surfaces of the OBs, respectively. Because the two homologous glomeruli are arranged symmetrically in the OB, odor information is represented and processed in two mirror maps (Mombaerts et al., 1996; Nagao et al., 2000, 2002; Zapiec and Mombaerts, 2015). The paired glomeruli are connected via axon collaterals of tufted cells in a point-to-point manner (Belluscio et al., 2002; Lodovichi et al., 2003; Marks et al., 2006). Although the anatomic arrangements of the two maps have been studied, less is known about the functional connections between them (Zhou and Belluscio, 2008, 2012). One pivotal idea is that the complex structure of the olfactory epithelium (OE) affects the sensitivity and timing of odorant responses of OSNs such that a delayed odorant response in one of the maps occurs with low odor concentrations (Kimbell et al., 1997; Schoenfeld and Cleland, 2006; Zhao et al., 2006; Zhou and Belluscio, 2012). The similarity in the spatiotemporal patterns representing odor information between these glomeruli remains unknown, partly because of the inaccessibility of the medial map, but is essential to understand the features that are common and uncommon between the two streams.

Trace amine-associated receptors (TAARs) were recently recognized as a second gene family of odorant receptors (Liberles and Buck, 2006). Pairs of OSNs expressing TAARs project to glomeruli in the mediodorsal OB (Ferrero et al., 2011; Pacifico et al., 2012; Dewan et al., 2013; Zhang et al., 2013; Liberles, 2015), which can frequently be observed simultaneously. Moreover, these two glomeruli are functionally identifiable because of their highly selective responses to the specific odorant at a low concentration (Zhang et al., 2013). In the present study, we measured simultaneous odorant responses of these homologous glomerular pairs and compared the response properties between medial and lateral maps. These glomeruli exhibited similar activity patterns of response onset latency, rise and decay times, and amplitudes. However, the medial glomeruli showed significantly larger respiration-locked fluctuations than the lateral glomeruli. The difference was observed in postsynaptic neuronal responses but not presynaptic terminal activity, suggesting that despite similar inputs, the medial map neurons and/or circuits enhance the respiration-locked activity for further odor information processing.

## Materials and Methods

All procedures were performed on mice of either sex in accordance with National Institutes of Health guidelines and approved by the Animal Welfare Committee at the University of Texas Health Science Center at Houston.

### Animals

Cre recombinase (Cre)-inducible GCaMP3-expressing mice (Ai38; #014538; RRID:IMSR_JAX:014538; Zariwala et al., 2012) were used for the expression of the calcium indicator in target neurons. This mouse line was crossed with the following Cre-driver mouse lines: OMP-Cre (for OSNs, #006668; RRID:IMSR_JAX:006668; Li et al., 2004), Gad2-Cre (for GABAergic neurons, #010802; RRID:IMSR_JAX:010802; Taniguchi et al., 2011), DAT-Cre (for dopaminergic neurons, #006660; RRID:IMSR_JAX:006660; Bäckman et al., 2006), and Pcdh21-Cre (for mitral/tufted cells, #O_2_,189; RRID:IMSR_RBRC02189; Nagai et al., 2005). Ai38, OMP-Cre, Gad2-Cre, and DAT-Cre mouse lines were purchased from The Jackson Laboratory. The Pcdh21-Cre mouse line was obtained from the RIKEN BioResource Research Center. All animals used in the experiments were aged between 8 and 16 weeks.

### Histology

Mice were deeply anesthetized and fixed by transcardial perfusion with 4% paraformaldehyde (PFA) in 0.1 M phosphate buffer (PB; pH 7.4). Then, whole brains were dissected out and postfixed in 4% PFA/0.1 M PB overnight. The samples were cryoprotected in 30% sucrose (wt/vol) in PBS (pH 7.4) and embedded in optimal cutting temperature compound (Fisher HealthCare). Olfactory tissue sections 30 μm thick were cut on a cryostat, washed with PBS, and mounted with Fluoroshield mounting medium (F6057; Sigma-Aldrich). Images were captured on an Olympus FluoView FV1000 laser scanning confocal microscope using a 20×/0.75 NA lens objective (UPLSAPO 20X; Olympus).

### Odorant stimulation

Isopentylamine (IPA; #126810; Sigma-Aldrich) and phenylethylamine (PEA; #128945; Sigma-Aldrich) were diluted in mineral oil (M3516; Sigma-Aldrich) to 0.1% and 0.01% in glass vials. The odorants were vaporized using nitrogen, mixed with filtered air to final concentrations of 0.02% and 0.002% with 0.5 l/min air flow rate, and then delivered to mouse nostrils using a custom-made olfactometer (Kikuta et al., 2013). The odorants were presented for 2 s with an interstimulus interval of >60 s to avoid sensory adaptation.

### *In vivo* optical imaging

GCaMP-expressing mice were anesthetized with urethane (1.2 g/kg of body weight, i.p.). The depth of anesthesia was monitored by toe pinches throughout the experiment. Body temperature was kept between 36.0°C and 37.0°C with a heating pad. The skull over the OBs was carefully thinned with a dental drill and covered with 1.2% agarose dissolved in saline and with a 4–6 mm × 4–6 mm^2^ coverslip (thickness, #1). Odor-evoked GCaMP3 signals were recorded through a 5× lens objective (Fluar 5×/0.25; Zeiss) on a microscope (SliceScope; Scientifica) equipped with a high-speed charge-coupled-device camera (NeuroCCD-SM256; RedShirtImaging) at 125 Hz (128 × 128 pixels) for 12 s, which included a 4-s prestimulus period and a 2-s odor presentation period. Excitation light was provided using a 470-nm light-emitting diode module (M470L2; Thorlabs). A standard green fluorescent protein filter set (BrightLine GFP-4050A-OMF-ZERO; Semrock) was used to detect the GCaMP3 signal. Chest movement of the animals was monitored to measure the respiratory rhythm during the optical imaging period.

### DiI labeling of OSN axons

After calcium imaging, the skull over the dorsal OBs was removed. Then, small DiI (1,1'-dioctadecyl-3,3,3'3'-tetramethylindocarbocyanine perchlorate) crystals were attached to the tip of glass capillaries (tip diameter, ∼5 μm) and embedded into the area of IPA-responsive glomeruli. The positions of blood vessels relative to homologous pairs of glomeruli were used as landmarks for DiI implantation. The mice remained anesthetized for 9–10 h after DiI implantation and then were fixed by transcardial perfusion with 4% PFA/0.1 M PB. Whole brains were removed and incubated in PBS at room temperature until observation. Low-magnification images were captured by a charge-coupled-device camera (NeuroCCD-SM256 or SensiCam; PCO) using a 5× lens objective (Fluar 5×/0.25, Zeiss) with a light-emitting diode module (MCWHL2-C1; Thorlabs) and a standard Cy3 cube (BrightLine Cy3-4040C-OMF-ZERO; Semrock). For high-magnification views of OSN axons, images were acquired with a two-photon microscope (Prairie Ultima), using a 20× water immersion lens objective (UMPLFLH 20XW; Olympus), with a 5-μm inter-z-slice interval and 512 × 512-pixel resolution. DiI was excited at 920 nm (Ti:sapphire laser, MaiTai HP DS; Spectra-Physics), and DiI fluorescence was detected with an emission filter cube (575-nm dichroic mirror and 607/45-nm barrier filters).

### Data analysis for wide-field calcium imaging data

Odor-evoked response maps were generated using Fiji/ImageJ (RRID:SCR_002285; Schindelin et al., 2012) with custom-written scripts. Spatially filtered (3 × 3 mean filter) prestimulation-period images (4 s) were averaged and used as a baseline (F_0_). Images averaged 3 s after the onset of the 1-s odor stimulation were used as the response image (F). Then, F was subtracted from F_0_ to obtain the difference (ΔF). ΔF values were divided by F_0_ to obtain the ratio image (ΔF/F_0_). Spatial filters (3 × 3 mean filter) were also applied to the ratio images. All negative values were set to zero in the images. Regions of interest corresponding to glomeruli were manually set (four to eight pixels centered on each glomerulus).

The time courses of calcium signals (Fig. 2*B*) were calculated using MATLAB (MathWorks; RRID:SCR_001622) with custom-written scripts. ΔF/F_0_ values were calculated using the same procedure described above but with a temporal filter (three frames average, 24 ms) rather than a spatial filter applied to the ΔF/F_0_ values.

Onset latency, rise time, decay time, and peak amplitude were computed with custom MATLAB scripts. First, the baseline values (ΔF/F_0_) were determined as the mean values over the baseline period, which was defined as a duration of 1 s during the prestimulation period. Then, the noise level in each trial was defined as the minimum standard deviation among eight 0.5-s blocks in the 4-s prestimulation period. The onset latency was determined as the first time point at which all data points in the subsequent 80 ms exceeded the threshold (2.5 times the noise level). The onset latency was measured as the time elapsed from the first inhalation after the onset of odor presentation. The rise time was defined as the duration the calcium signals increased from 20% to 80% of peak amplitude. Time points for when the calcium signals reached 20% and 80% of the peak amplitude were set as the earliest time point after which half of the data points in the subsequent 80 ms exceeded these criteria. The decay time was defined as the duration the calcium signal decreased from 100% to 50% of the peak amplitude. Time points for when the calcium signals reached 100% and 50% of peak amplitude were set as the earliest time point after which half of data points in the subsequent 80 ms dropped below these criteria. The peak amplitude was measured as the maximum value using the 80-ms time window moving average, which reflects an average of nine sequential data points, after stimulation onset.

To analyze fluctuations in the calcium fluorescence, a power spectral analysis based on Fourier transform was used. First, ΔF/F_0_ values were preprocessed with a 40-ms box filter and divided into two periods, corresponding to prestimulation (2 s before onset of odor stimulation) and odor stimulation (4 s after onset of odor stimulation). Then, a power spectrum was computed by using Fast Fourier transform with 2048 points [using a built-in function of MATLAB (fft.m)] in each period. Because we used a 125-Hz sampling frequency, the frequency resolution is 0.061 Hz. The peak power in each period was determined as the maximum value between 2 and 4 Hz. The background baseline was defined as the mean value of the power spectrum between 6 and 9 Hz and was subtracted from the spectrum.

### Experimental design and statistical analyses

Experiments were designed to simultaneously record odor-evoked responses from homologous glomerular pairs and to compare the response properties between the medial and lateral maps as described above.

Statistical analyses were performed using Microsoft Excel 2013. All statistical significance was determined by a two-tailed paired Student’s *t* test; *p* < 0.05 was considered statistically significant. Data are presented as scatter and box-whisker plots of pooled datasets for a given odorant and concentration from OMP-Cre (279 trials in 0.02% PEA, 194 trials in 0.002% PEA, 297 trials in 0.02% IPA, and 185 trials in 0.002% IPA; *n *=* *10), Gad2-Cre (106 trials in 0.02% PEA, 84 trials in 0.002% PEA, 119 trials in 0.02% IPA, and 62 trials in 0.002% IPA; *n *=* *6), DAT-Cre (216 trials in 0.02% PEA, 118 trials in 0.002% PEA, 184 trials in 0.02% IPA, and 123 trials in 0.002% IPA; *n *=* *6), and Pcdh21-Cre (199 trials in 0.02% PEA, 130 trials in 0.002% PEA, 241 trials in 0.02% IPA, and 176 trials in 0.002% IPA; *n *=* *6) mice. In scatter plots, individual dots show data points from single trials. In box-whisker plots, horizontal red lines and boxes indicate the medians and quartiles, respectively. The whiskers go from each quartile to the minimum or maximum. All statistical data (sample size, 95% confidence interval, mean, standard deviation, and *p* values) are listed in Table 2.

## Results

### Expression patterns of GCaMP3 in OBs from Cre mouse driver lines

**Figure 1.**
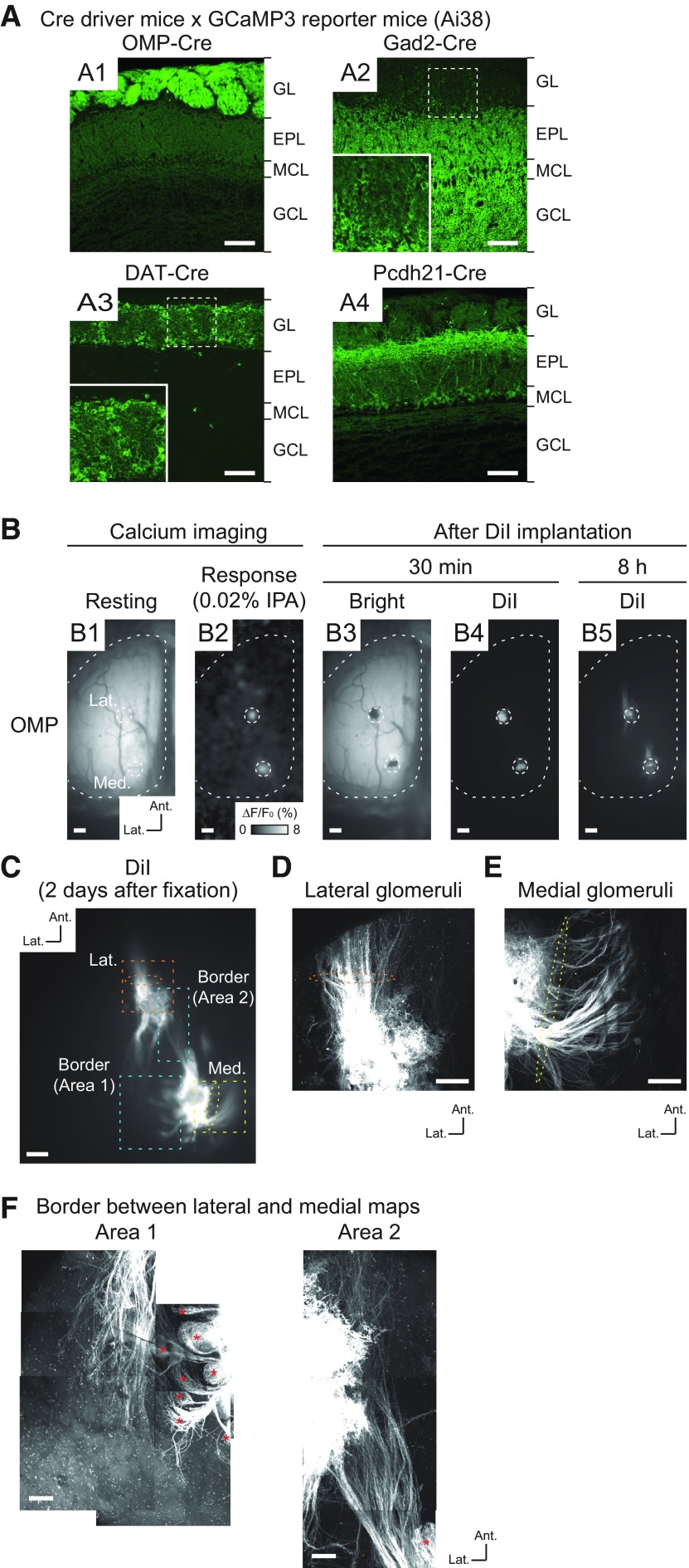
Cell-type-specific expression of GCaMP3 in OBs and distinct OSN axon trajectories in IPA-responsive glomeruli. ***A1–A4***, Confocal images of OBs in Cre-dependent GCaMP3 reporter mice crossed with OMP-Cre (***A1***), Gad2-Cre (***A2***), DAT-Cre (***A3***), and Pcdh21-Cre (***A4***) mice. Magnified views of the dashed squares are shown in the insets in ***A2***, ***A3***. GL, EPL, MCL, and GCL indicate GL, external plexiform layer, MCL, and GCL, respectively. ***B***, Process for the DiI labeling of OSN axons. ***B1***, Resting fluorescence of GCaMP3 in the dorsal OB of OMP-Cre mouse. ***B2***, IPA (0.02%)-responsive homologous glomeruli were observed by calcium imaging [color scale indicates ΔF/F_0_ (%) of GCaMP3 signal]. ***B3***, Brightfield image after DiI implantation. ***B4***, DiI fluorescence 30 min after DiI implantation. ***B5***, DiI fluorescence 8 h after DiI implantation. The locations of IPA-responsive glomeruli are indicated by the white dotted circles. ***C***, Two-photon microscopy image of DiI-labeled glomeruli and OSN axons 2 d after DiI implantation. ***D***, Magnified image of area denoted by orange dotted square in ***C*** associated with lateral glomerulus. ***E***, Magnified image of area denoted by yellow dotted square in ***C*** associated with medial glomerulus. Two-photon microscopy images of OSN axons that transverse the lateral and medial surface of OB; orange and yellow dotted ellipses (in ***C*–*E***) represent major axonal projections from lateral and medial glomeruli. ***F***, Two-photon microscopy images of areas of lateral/medial border denoted by blue dotted square in ***C***. Red asterisks indicate axon termination in several glomeruli in which the labeled OSNs probably passed through the surface of the DiI-implanted glomeruli. Some minor axons which did not show clear axon terminations in a glomerulus were observed in area 2 in this case. Ant., anterior. Lat., lateral. Scale bars: 100 μm (***A***), 200 μm (***B***, ***C***), 50 μm (***D***, ***E***), and 100 μm (***F***).

We recorded odor-evoked neuronal activity in the OBs of mice from multiple transgenic mouse lines. To express the genetically encoded calcium indicator GCaMP3 (Tian et al., 2009) in different types of neurons in the OB, we used four Cre-driver mouse lines. Specifically, OMP-Cre (Li et al., 2004), Gad2-Cre (Taniguchi et al., 2011), DAT-Cre (Bäckman et al., 2006), and Pcdh21-Cre (Nagai et al., 2005) mouse lines were crossed with a Cre-inducible GCaMP3 reporter mouse line (Ai38; Zariwala et al., 2012) so that GCaMP3 was expressed in OSNs and GABAergic, dopaminergic, and mitral/tufted cells, respectively. We first verified the GCaMP3 expression pattern in each of the Cre-driver mouse lines (Fig. 1*A1–A4*). In OMP-Cre mice, GCaMP3 signals were clearly detected only in OSNs in the glomerular layer (GL; Fig. 1*A1*). Consistent with a previous report (Wachowiak et al., 2013), GCaMP3 in Gad2-Cre mice was strongly expressed in the external plexiform layer (EPL) and in the granule cell layer (GCL); the expression was predominantly by granule cells, but enhanced detection methods also revealed expression by periglomerular cells in the GCL (Fig. 1*A2*). In DAT-Cre mice, GCaMP3 signals were mostly restricted to the GL (Fig. 1*A3*). High magnification of this layer indicated that GCaMP3 was expressed by juxtaglomerular cells, which were considered to be short-axon (SA) cells (Kiyokage et al., 2010; Fig. 1*A3*, inset). This pattern of expression was similar to that in the tyrosine hydroxylase (TH)-Cre line, another driver line for expression in dopaminergic cells (Wachowiak et al., 2013). In Pcdh21-Cre mice, GCaMP3 was specifically expressed in mitral/tufted cells, as GCaMP3 signals appeared in somata and neurite processes in superficial EPL and the mitral cell layer (MCL; Fig. 1*A4*), as reported previously (Nagai et al., 2005; Mizuguchi et al., 2012; Huang et al., 2013). Although reporter expression is induced in OSNs in another Pcdh21-Cre-driver line (Wachowiak et al., 2013), we did not observe this ectopic expression in our mice. In summary, these Cre mouse lines exhibited the expected cell-type-specific GCaMP3 expression patterns.

**Figure 2.**
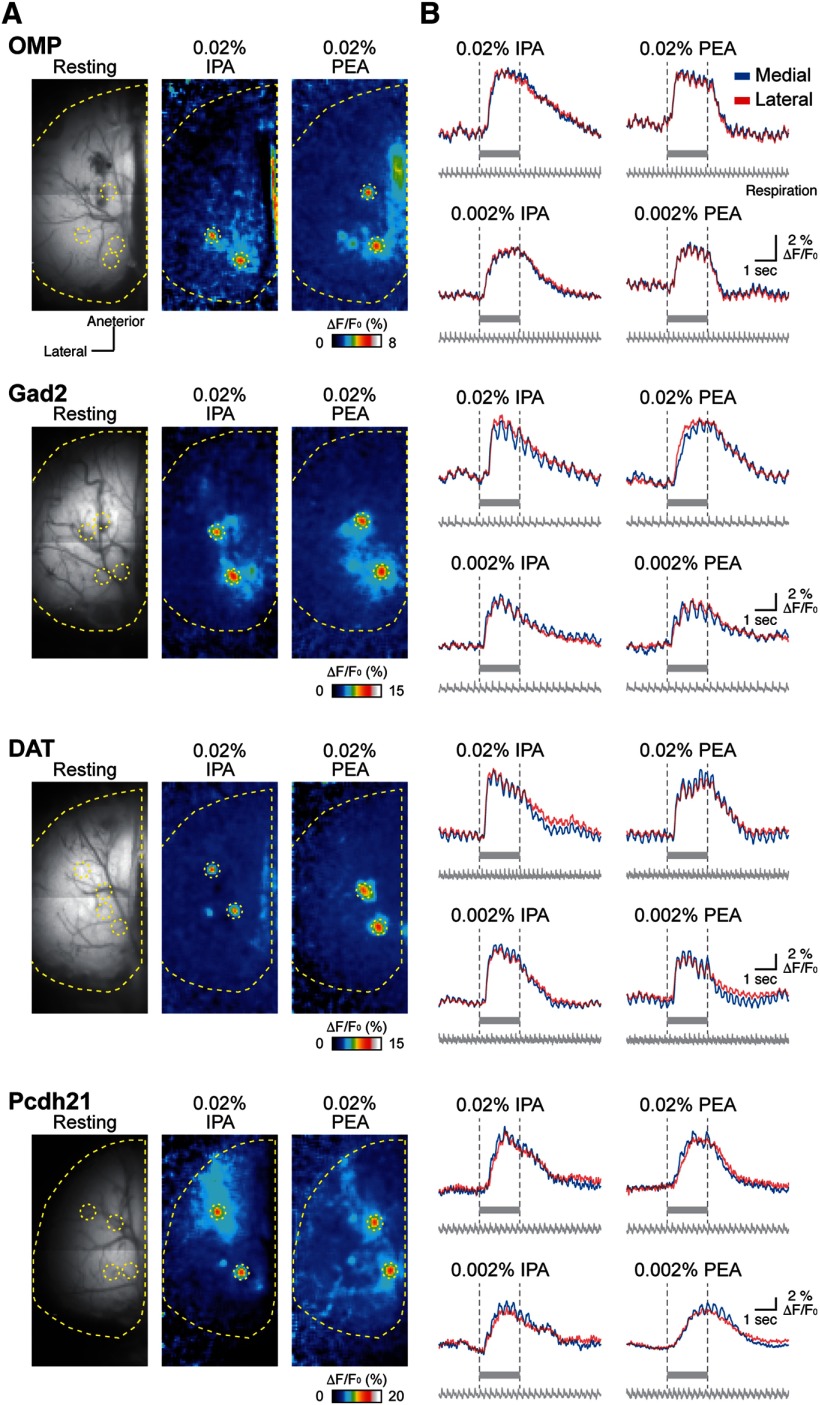
Odor-evoked response maps and traces among different types of OB neurons. Odor-evoked response maps (***A***) and traces (***B***) from OMP-Cre, Gad2-Cre, DAT-Cre, and Pcdh21-Cre mice. Resting GCaMP3 fluorescence images are displayed in the left panels in ***A***. Pseudocolored images in the middle and right panels in ***A*** indicate responses to 0.02% IPA and PEA, respectively. Yellow dotted lines and circles in each image show approximately the edge of the left OB and homologous glomeruli evoked by 0.02% IPA and PEA, respectively. The color scales represent ΔF/F_0_ (%) of GCaMP3 signal. Traces shown in B represent GCaMP3 fluorescence changes from two subsets of homologous glomeruli evoked by indicated concentrations of IPA and PEA shown in A. Red and blue traces indicate lateral and medial glomeruli, respectively. Gray bars under each trace and vertical dotted lines indicate the timing of odor stimulation. Respiration signals are also shown under the traces.

**Figure 3.**
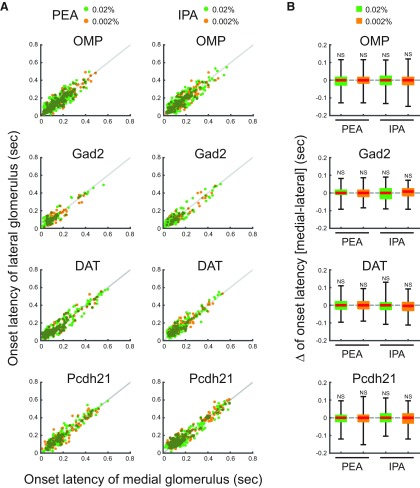
Onset latencies of medial and lateral glomeruli. ***A***, Scatter plots displaying the distributions of onset latencies of medial and lateral glomeruli, which responded to PEA and IPA stimuli. *x* and *y* axes indicate onset latencies of medial and lateral glomerular responses, respectively. Individual green and orange dots indicate single trial data of 0.02% and 0.002% PEA or IPA. Gray lines indicate the equal onset latency time points of medial and lateral glomerular responses. ***B***, Box plots displaying the distributions of the differences of onset latencies between medial and lateral glomeruli responses to 0.02% and 0.002% PEA or IPA. Red horizontal lines in each box indicate the medians. Quartiles are shown as whiskers. NS, not significant (two-tailed paired Student’s *t* test).

**Figure 4.**
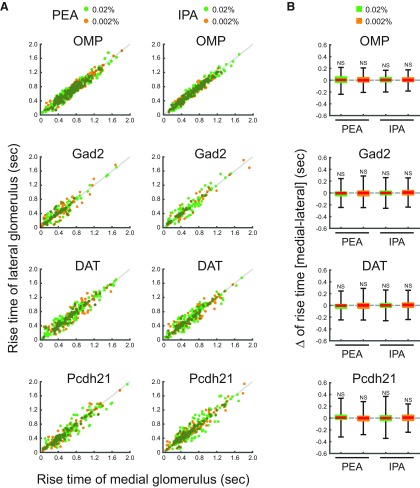
Rise times of medial and lateral glomeruli. ***A***, Scatter plots displaying the distributions of rise times of the medial and lateral glomeruli, which responded to PEA and IPA stimuli. *x* and *y* axes indicate rise times of medial and lateral glomerular responses, respectively. Individual green and orange dots indicate single trial data of 0.02% and 0.002% PEA or IPA. Gray lines indicate the equal rise time points of medial and lateral glomerular responses. ***B***, Box plots displaying the distributions of the differences of rise times between medial and lateral glomerular responses to 0.02% and 0.002% PEA or IPA. Red horizontal lines in each box indicate the medians. Quartiles are shown as whiskers. NS, not significant (two-tailed paired Student’s *t* test).

### Identification of homologous glomeruli in medial and lateral maps

*In vivo* optical imaging of the dorsal OB has not been used as a means to record activity in the medial map, because most of the glomeruli are located in the medioventral region of the OB (Johnson et al., 1995, 1999, 2004, 2005, 2009; Johnson and Leon, 1996, 2000; Nagao et al., 2000, 2002; Inaki et al., 2002; Taniguchi et al., 2003; Mori et al., 2006; Zapiec and Mombaerts, 2015). However, it was recently demonstrated that OSNs expressing TAARs project to two or a few glomeruli in caudal regions of the dorsal OB (Johnson et al., 2012; Pacifico et al., 2012; Dewan et al., 2013, 2018; Zhang et al., 2013; Liberles, 2015). These medial and lateral glomeruli can easily be identified, because axons from TAAR-expressing OSNs projecting to the dorsomedial glomeruli transverse the anteromedial (septal) surface of the OB, whereas those projecting to the dorsolateral glomeruli transverse the anterolateral surface. The axonal trajectories and odorant response profiles of glomeruli receiving projections from OSNs expressing TAAR3 and TAAR4 are well characterized (Pacifico et al., 2012; Dewan et al., 2013, 2018; Zhang et al., 2013). Specifically, these glomeruli are highly sensitive to IPA and PEA, respectively. Therefore, we imaged simultaneously these medial and lateral glomeruli that receive input from TAAR3-expressing and TAAR4-expressing OSNs.

Using IPA and PEA at final concentrations of 0.02% and 0.002%, which reliably and specifically induce activation of the glomeruli receiving inputs from TAAR3-expressing and TAAR4-expressing OSNs, respectively (Pacifico et al., 2012; Dewan et al., 2013, 2018; Zhang et al., 2013), we identified distinct pairs of glomeruli comprising OSNs and GABAergic, dopaminergic, and mitral/tufted cells in the caudal areas of the dorsal OB (Figs. 1*B*, 2*A*). The locations of these homologous glomeruli are consistent with the positions of glomeruli receiving inputs from TAAR3-expressing and TAAR4-expressing neurons in previous reports (Pacifico et al., 2012; Dewan et al., 2013, 2018; Zhang et al., 2013).

To confirm that the glomeruli pairs represent medial and lateral maps, we retrogradely labeled TAAR3-expressing OSN axons by implanting the IPA-responsive glomeruli in OMP-GCaMP3 mice with DiI crystals (*n* = 3). Optical observation performed 8 h later revealed labeling of OSN axons in both lateral and medial glomeruli (Fig. 1*B5*). These labeled axons were observed more clearly in fixed tissue 2 d later (Fig. 1*C*). Dorsolateral to the lateral IPA-responsive glomerulus, the majority of the labeled axons were oriented in an anterolateral direction (Fig. 1*C*,*D*, orange dashed ellipses). By contrast, the majority of axons dorsomedial to the medial IPA-responsive glomerulus were in an anteromedial direction (toward the septum; Fig. 1*C*,*E*, yellow dotted ellipses). Notably, multiple glomerular structures were revealed by the terminal branches of labeled axons in the medial region (Fig. 1*F*, red asterisks), indicating that these axons originated from the medial side. Taken together, these results indicate that the pairs of recorded glomeruli were homologous pairs representing both lateral and medial maps. Therefore, our experimental design provides a unique opportunity to record simultaneously the odor-evoked neuronal activity of homologous glomeruli in the medial and lateral maps in OBs.

**Figure 5.**
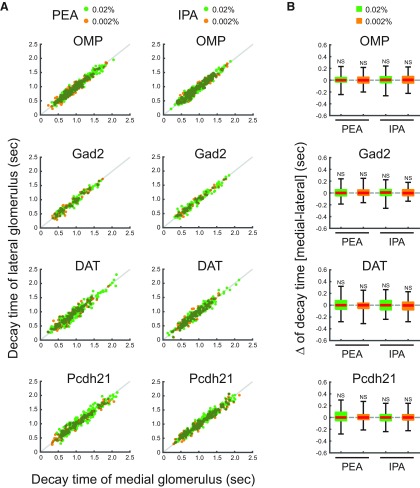
Decay times of medial and lateral glomeruli. ***A***, Scatter plots displaying the distributions of decay times of the medial and lateral glomeruli, which responded to PEA and IPA stimuli. *x* and *y* axes indicate decay times of medial and lateral glomerular responses, respectively. Individual green and orange dots indicate single trial data of 0.02% and 0.002% PEA or IPA. Gray lines indicate the equal decay time points of medial and lateral glomerular responses. ***B***, Box plots displaying the distributions of the differences of decay times between medial and lateral glomerular responses to 0.02% and 0.002% PEA or IPA. Red horizontal lines in each box indicate the medians. Quartiles are shown as whiskers. NS, not significant (two-tailed paired Student’s *t* test).

### Similar temporal odor representations between medial and lateral maps in the OB

**Figure 6.**
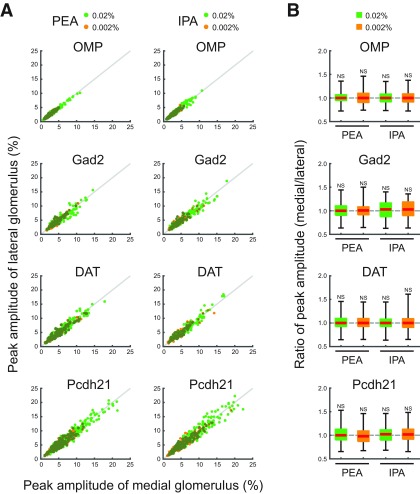
Peak amplitude of medial and lateral glomerular responses. ***A***, Scatter plots displaying the distributions of peak amplitudes of medial and lateral glomerular responses to PEA and IPA stimuli. *x-* and *y*-axes indicate peak amplitudes of medial and lateral glomerular responses, respectively. Individual green and orange dots indicate single trial data of 0.02% and 0.002% PEA or IPA. Gray lines indicate the equal peak amplitudes of medial and lateral glomerular responses. ***B***, Box plots displaying the distributions of the differences of peak amplitudes of medial and lateral glomerular responses to 0.02% and 0.002% PEA or IPA. Red horizontal lines in each box indicate the medians. Quartiles are shown as whiskers. NS, not significant (two-tailed paired Student’s *t* test).

The medial and lateral glomeruli pairs represent inputs from odorant receptors of the same type within medial and lateral regions, respectively, of the complex OE structure. Receptors in these regions may be exposed to different air flow rates and mucosal volumes. We hypothesized that these differences would be reflected in the timing of the glomeruli responses. However, the calcium signals from all cell types examined in medial and lateral glomeruli in response to IPA and PEA had similar amplitudes and temporal patterns (Fig. 2*B*). Further analyses revealed that the timing of odor inputs to both maps was similar, as revealed by the onset latency measured as the time at which the calcium signal exceeded the threshold from first inhalation during odor stimulation (Fig. 3). The rise times of the responses, which are an indicator of response speed and reflect the neuronal spike frequency, were similar between medial and lateral glomeruli (Fig. 4). The rise time was assessed as the duration for the calcium signal to increase from 20% to 80% of the peak signal. Conversely, the similar decay times, during which the calcium signal decreased from 100% to 50% of the peak signals, suggested that the activity in one glomerulus was not prolonged relative to the other after the odor stimulus was turned off (Fig. 5). Both glomeruli in the pairs had responses that were similar in strength, as indicated by the peak amplitudes (Fig. 6). The results obtained under all experimental conditions are summarized in Table 1. Overall, there were no significant differences between paired glomeruli in onset latency, rise time, decay time, and peak amplitude of the calcium responses in any of the cell types studied (two-tailed paired *t* tests; Table 2).

**Table 1:** Average and error values in each experimental condition

Figure	Mice	Odorant	Conc, %	*N*	Mean (medial), s	SD (medial), s	Mean (lateral), s	SD (lateral), s
3*A*	OMP	PEA	0.02	279	0.138	0.086	0.142	0.086
3*A*	OMP	PEA	0.002	194	0.153	0.096	0.153	0.098
3*A*	OMP	IPA	0.02	297	0.151	0.101	0.154	0.100
3*A*	OMP	IPA	0.002	185	0.155	0.095	0.158	0.097
3*A*	Gad2	PEA	0.02	106	0.103	0.099	0.108	0.096
3*A*	Gad2	PEA	0.002	84	0.125	0.083	0.128	0.081
3*A*	Gad2	IPA	0.02	119	0.112	0.110	0.113	0.104
3*A*	Gad2	IPA	0.002	62	0.143	0.105	0.146	0.101
3*A*	DAT	PEA	0.02	216	0.157	0.132	0.158	0.127
3*A*	DAT	PEA	0.002	118	0.162	0.128	0.162	0.125
3*A*	DAT	IPA	0.02	184	0.148	0.096	0.152	0.098
3*A*	DAT	IPA	0.002	123	0.160	0.098	0.166	0.093
3*A*	Pcdh21	PEA	0.02	199	0.183	0.125	0.185	0.129
3*A*	Pcdh21	PEA	0.002	130	0.197	0.126	0.202	0.126
3*A*	Pcdh21	IPA	0.02	241	0.179	0.128	0.178	0.123
3*A*	Pcdh21	IPA	0.002	176	0.210	0.147	0.214	0.144
4*A*	OMP	PEA	0.02	279	0.638	0.305	0.631	0.300
4*A*	OMP	PEA	0.002	194	0.732	0.307	0.724	0.307
4*A*	OMP	IPA	0.02	297	0.658	0.300	0.653	0.304
4*A*	OMP	IPA	0.002	185	0.679	0.279	0.674	0.275
4*A*	Gad2	PEA	0.02	106	0.425	0.302	0.441	0.300
4*A*	Gad2	PEA	0.002	84	0.489	0.290	0.490	0.297
4*A*	Gad2	IPA	0.02	119	0.486	0.301	0.496	0.314
4*A*	Gad2	IPA	0.002	62	0.549	0.377	0.543	0.381
4*A*	DAT	PEA	0.02	216	0.651	0.357	0.656	0.352
4*A*	DAT	PEA	0.002	118	0.656	0.324	0.654	0.328
4*A*	DAT	IPA	0.02	184	0.594	0.310	0.606	0.308
4*A*	DAT	IPA	0.002	123	0.597	0.311	0.602	0.301
4*A*	Pcdh21	PEA	0.02	199	0.590	0.327	0.580	0.321
4*A*	Pcdh21	PEA	0.002	130	0.521	0.326	0.518	0.330
4*A*	Pcdh21	IPA	0.02	241	0.590	0.313	0.585	0.327
4*A*	Pcdh21	IPA	0.002	176	0.586	0.332	0.579	0.330
5*A*	OMP	PEA	0.02	279	1.007	0.356	1.003	0.358
5*A*	OMP	PEA	0.002	194	0.883	0.297	0.879	0.301
5*A*	OMP	IPA	0.02	297	0.939	0.306	0.934	0.313
5*A*	OMP	IPA	0.002	185	0.945	0.335	0.940	0.327
5*A*	Gad2	PEA	0.02	106	0.890	0.340	0.888	0.337
5*A*	Gad2	PEA	0.002	84	0.858	0.346	0.857	0.349
5*A*	Gad2	IPA	0.02	119	0.917	0.365	0.909	0.358
5*A*	Gad2	IPA	0.002	62	0.846	0.349	0.845	0.352
5*A*	DAT	PEA	0.02	216	0.936	0.386	0.930	0.388
5*A*	DAT	PEA	0.002	118	0.850	0.316	0.853	0.311
5*A*	DAT	IPA	0.02	184	0.926	0.356	0.928	0.361
5*A*	DAT	IPA	0.002	123	0.839	0.295	0.849	0.301
5*A*	Pcdh21	PEA	0.02	199	1.101	0.412	1.098	0.400
5*A*	Pcdh21	PEA	0.002	130	0.975	0.416	0.969	0.393
5*A*	Pcdh21	IPA	0.02	241	1.083	0.399	1.088	0.396
5*A*	Pcdh21	IPA	0.002	176	1.041	0.407	1.032	0.392
6*A*	OMP	PEA	0.02	279	3.114	1.635	3.099	1.597
6*A*	OMP	PEA	0.002	194	2.244	0.963	2.248	0.979
6*A*	OMP	IPA	0.02	297	3.096	1.741	3.123	1.772
6*A*	OMP	IPA	0.002	185	2.469	1.078	2.478	1.077
6*A*	Gad2	PEA	0.02	106	5.910	2.435	5.869	2.360
6*A*	Gad2	PEA	0.002	84	4.309	1.712	4.349	1.831
6*A*	Gad2	IPA	0.02	119	6.442	3.217	6.294	3.029
6*A*	Gad2	IPA	0.002	62	4.695	1.982	4.626	1.950
6*A*	DAT	PEA	0.02	216	5.895	2.866	5.870	2.763
6*A*	DAT	PEA	0.002	118	4.739	2.480	4.693	2.312
6*A*	DAT	IPA	0.02	184	5.851	2.892	5.877	2.991
6*A*	DAT	IPA	0.002	123	4.266	2.174	4.290	2.142
6*A*	Pcdh21	PEA	0.02	199	7.565	4.335	7.531	4.281
6*A*	Pcdh21	PEA	0.002	130	5.506	2.356	5.601	2.302
6*A*	Pcdh21	IPA	0.02	241	7.610	4.344	7.576	4.301
6*A*	Pcdh21	IPA	0.002	176	5.532	2.858	5.444	2.744

**Table 2:** Summary of statistical analyses

Figure	Mice	Odorant	Conc, %	*N*	Lower 95% CI, ms	Upper 95% CI, ms	Mean, ms	SD, ms	*p*
3*B*	OMP	PEA	0.02	279	–9.6	1.2	−4.2	45.9	0.130
3*B*	OMP	PEA	0.002	194	−6.0	5.0	−0.5	38.8	0.853
3*B*	OMP	IPA	0.02	297	−9.3	1.7	−3.8	48.2	0.172
3*B*	OMP	IPA	0.002	185	−8.8	3.1	−2.8	41.0	0.346
3*B*	Gad2	PEA	0.02	106	−12.2	1.1	−5.5	34.6	0.101
3*B*	Gad2	PEA	0.002	84	−10.8	5.7	−2.5	38.2	0.542
3*B*	Gad2	IPA	0.02	119	−8.5	7.1	−0.7	43.2	0.862
3*B*	Gad2	IPA	0.002	62	−13.0	7.4	−2.8	40.1	0.582
3*B*	DAT	PEA	0.02	216	−6.0	5.3	−0.3	42.3	0.905
3*B*	DAT	PEA	0.002	118	−7.7	7.9	0.1	42.7	0.983
3*B*	DAT	IPA	0.02	184	−10.0	1.0	−4.5	37.9	0.111
3*B*	DAT	IPA	0.002	123	−13.1	0.8	−6.2	38.9	0.080
3*B*	Pcdh21	PEA	0.02	199	−7.2	3.1	−2.1	36.8	0.428
3*B*	Pcdh21	PEA	0.002	130	−12.7	2.4	−5.1	43.6	0.182
3*B*	Pcdh21	IPA	0.02	241	−4.1	6.9	1.4	43.1	0.610
3*B*	Pcdh21	IPA	0.002	176	−10.8	1.9	−4.4	42.4	0.166
4*B*	OMP	PEA	0.02	279	−2.1	15.8	6.9	76.1	0.133
4*B*	OMP	PEA	0.002	194	−3.5	18.8	7.6	78.7	0.178
4*B*	OMP	IPA	0.02	297	−2.6	11.9	4.7	63.6	0.207
4*B*	OMP	IPA	0.002	185	−3.7	14.5	5.4	62.7	0.245
4*B*	Gad2	PEA	0.02	106	−36.1	3.1	−16.5	101.7	0.098
4*B*	Gad2	PEA	0.002	84	−26.5	24.0	−1.2	116.2	0.922
4*B*	Gad2	IPA	0.02	119	−28.6	7.7	−10.4	99.9	0.257
4*B*	Gad2	IPA	0.002	62	−16.9	30.0	6.6	92.3	0.577
4*B*	DAT	PEA	0.02	216	−17.6	9.5	−4.1	100.7	0.555
4*B*	DAT	PEA	0.002	118	−19.5	22.3	1.4	114.5	0.893
4*B*	DAT	IPA	0.02	184	−27.3	3.0	−12.2	103.5	0.116
4*B*	DAT	IPA	0.002	123	−23.2	12.2	−5.5	100.5	0.541
4*B*	Pcdh21	PEA	0.02	199	−4.3	24.7	10.2	103.7	0.165
4*B*	Pcdh21	PEA	0.002	130	−12.9	17.9	2.5	88.7	0.745
4*B*	Pcdh21	IPA	0.02	241	−10.0	20.2	5.1	119.0	0.504
4*B*	Pcdh21	IPA	0.002	176	−5.8	20.8	7.5	89.2	0.267
5*B*	OMP	PEA	0.02	279	−5.1	12.3	3.6	74.2	0.419
5*B*	OMP	PEA	0.002	194	−8.6	15.2	3.3	83.9	0.583
5*B*	OMP	IPA	0.02	297	−4.2	14.2	5.0	80.8	0.289
5*B*	OMP	IPA	0.002	185	−8.3	16.4	4.1	85.3	0.517
5*B*	Gad2	PEA	0.02	106	−12.9	17.6	2.4	79.2	0.757
5*B*	Gad2	PEA	0.002	84	−15.5	18.3	1.4	77.8	0.867
5*B*	Gad2	IPA	0.02	119	−6.6	22.9	8.2	81.4	0.276
5*B*	Gad2	IPA	0.002	62	−15.5	18.3	1.4	66.6	0.869
5*B*	DAT	PEA	0.02	216	−8.6	21.2	6.3	111.0	0.407
5*B*	DAT	PEA	0.002	118	−21.7	15.9	−2.9	103.1	0.757
5*B*	DAT	IPA	0.02	184	−16.4	12.5	−2.0	99.4	0.789
5*B*	DAT	IPA	0.002	123	−27.8	6.3	−10.7	95.5	0.215
5*B*	Pcdh21	PEA	0.02	199	−12.5	19.8	3.6	115.7	0.659
5*B*	Pcdh21	PEA	0.002	130	−6.9	19.7	6.4	76.6	0.345
5*B*	Pcdh21	IPA	0.02	241	−16.5	5.7	−5.4	88.2	0.337
5*B*	Pcdh21	IPA	0.002	176	−3.7	21.4	8.8	82.9	0.166
6*B*	OMP	PEA	0.02	279	0.998	1.025	1.012	0.115	0.385
6*B*	OMP	PEA	0.002	194	0.994	1.034	1.014	0.142	0.864
6*B*	OMP	IPA	0.02	297	0.987	1.016	1.002	0.127	0.274
6*B*	OMP	IPA	0.002	185	0.985	1.023	1.004	0.133	0.708
6*B*	Gad2	PEA	0.02	106	0.985	1.044	1.014	0.173	0.629
6*B*	Gad2	PEA	0.002	84	0.982	1.047	1.014	0.166	0.531
6*B*	Gad2	IPA	0.02	119	0.998	1.056	1.027	0.181	0.077
6*B*	Gad2	IPA	0.002	62	0.994	1.077	1.035	0.189	0.479
6*B*	DAT	PEA	0.02	216	0.991	1.028	1.009	0.150	0.646
6*B*	DAT	PEA	0.002	118	0.983	1.039	1.011	0.167	0.472
6*B*	DAT	IPA	0.02	184	0.985	1.024	1.005	0.143	0.624
6*B*	DAT	IPA	0.002	123	0.979	1.033	1.006	0.166	0.680
6*B*	Pcdh21	PEA	0.02	199	0.996	1.041	1.019	0.178	0.648
6*B*	Pcdh21	PEA	0.002	130	0.963	1.020	0.992	0.180	0.216
6*B*	Pcdh21	IPA	0.02	241	0.997	1.037	1.017	0.170	0.652
6*B*	Pcdh21	IPA	0.002	176	1.003	1.050	1.026	0.172	0.159
7*C* Pre	OMP	PEA	0.02	279	0.984	1.011	0.998	0.111	0.703
7*C* Pre	OMP	PEA	0.002	194	0.980	1.016	0.998	0.129	0.834
7*C* Pre	OMP	IPA	0.02	297	0.997	1.026	1.011	0.128	0.135
7*C* Pre	OMP	IPA	0.002	185	0.992	1.030	1.011	0.122	0.253
7*C* Pre	Gad2	PEA	0.02	106	1.300	1.407	1.354	0.289	4.011 E–24
7*C* Pre	Gad2	PEA	0.002	84	1.270	1.389	1.330	0.282	1.935 E–18
7*C* Pre	Gad2	IPA	0.02	119	1.296	1.407	1.351	0.322	4.059 E–24
7*C* Pre	Gad2	IPA	0.002	62	1.304	1.449	1.376	0.315	4.941 E–16
7*C* Pre	DAT	PEA	0.02	216	1.258	1.323	1.291	0.245	3.173 E–43
7*C* Pre	DAT	PEA	0.002	118	1.220	1.304	1.262	0.230	8.205 E–23
7*C* Pre	DAT	IPA	0.02	184	1.222	1.287	1.255	0.226	1.910 E–35
7*C* Pre	DAT	IPA	0.002	123	1.201	1.302	1.251	0.276	6.661 E–17
7*C* Pre	Pcdh21	PEA	0.02	199	1.264	1.351	1.308	0.339	1.512 E–32
7*C* Pre	Pcdh21	PEA	0.002	130	1.247	1.347	1.297	0.300	1.512 E–22
7*C* Pre	Pcdh21	IPA	0.02	241	1.292	1.357	1.325	0.273	6.790 E–55
7*C* Pre	Pcdh21	IPA	0.002	176	1.281	1.355	1.318	0.258	1.024 E–39
7*C* Odor	OMP	PEA	0.02	279	0.990	1.021	1.005	0.131	0.492
7*C* Odor	OMP	PEA	0.002	194	0.997	1.032	1.015	0.128	0.104
7*C* Odor	OMP	IPA	0.02	297	0.988	1.019	1.004	0.135	0.635
7*C* Odor	OMP	IPA	0.002	185	0.984	1.021	1.002	0.120	0.812
7*C* Odor	Gad2	PEA	0.02	106	1.475	1.630	1.552	0.415	1.008 E–26
7*C* Odor	Gad2	PEA	0.002	84	1.506	1.693	1.600	0.448	1.009 E–21
7*C* Odor	Gad2	IPA	0.02	119	1.508	1.670	1.589	0.469	7.588 E–29
7*C* Odor	Gad2	IPA	0.002	62	1.491	1.652	1.571	0.350	9.631 E–23
7*C* Odor	DAT	PEA	0.02	216	1.405	1.495	1.450	0.338	7.929 E–50
7*C* Odor	DAT	PEA	0.002	118	1.424	1.564	1.494	0.382	1.060 E–26
7*C* Odor	DAT	IPA	0.02	184	1.413	1.522	1.467	0.382	2.147 E–39
7*C* Odor	DAT	IPA	0.002	123	1.407	1.555	1.481	0.400	3.559 E–24
7*C* Odor	Pcdh21	PEA	0.02	199	1.416	1.507	1.462	0.351	5.482 E–53
7*C* Odor	Pcdh21	PEA	0.002	130	1.460	1.595	1.528	0.405	6.719 E–32
7*C* Odor	Pcdh21	IPA	0.02	241	1.435	1.529	1.482	0.397	2.219 E–56
7*C* Odor	Pcdh21	IPA	0.002	176	1.464	1.587	1.526	0.427	1.386 E–39
7*D*	OMP	PEA	0.02	558	1.347	1.421	1.384	0.454	3.053 E–05
7*D*	OMP	PEA	0.002	388	1.413	1.496	1.454	0.394	0.017
7*D*	OMP	IPA	0.02	594	1.371	1.434	1.403	0.388	9.829 E–14
7*D*	OMP	IPA	0.002	370	1.275	1.353	1.314	0.359	3.323 E–06
7*D*	Gad2	PEA	0.02	212	1.395	1.484	1.440	0.340	1.917 E–04
7*D*	Gad2	PEA	0.002	168	1.402	1.514	1.458	0.384	1.931 E–04
7*D*	Gad2	IPA	0.02	238	1.404	1.496	1.450	0.379	2.091 E–04
7*D*	Gad2	IPA	0.002	124	1.415	1.523	1.469	0.335	1.401 E–03
7*D*	DAT	PEA	0.02	432	1.338	1.393	1.365	0.292	8.842 E–06
7*D*	DAT	PEA	0.002	236	1.327	1.403	1.365	0.295	4.632 E–05
7*D*	DAT	IPA	0.02	368	1.319	1.379	1.349	0.294	8.927 E–05
7*D*	DAT	IPA	0.002	246	1.307	1.387	1.347	0.309	2.816 E–04
7*D*	Pcdh21	PEA	0.02	398	1.348	1.409	1.378	0.339	8.010 E–03
7*D*	Pcdh21	PEA	0.002	260	1.360	1.441	1.400	0.344	5.501 E–03
7*D*	Pcdh21	IPA	0.02	482	1.363	1.416	1.390	0.314	3.800 E–03
7*D*	Pcdh21	IPA	0.002	352	1.369	1.433	1.401	0.316	8.931 E–04

### Respiration-locked calcium fluctuations in medial maps are larger than in lateral maps

**Figure 7.**
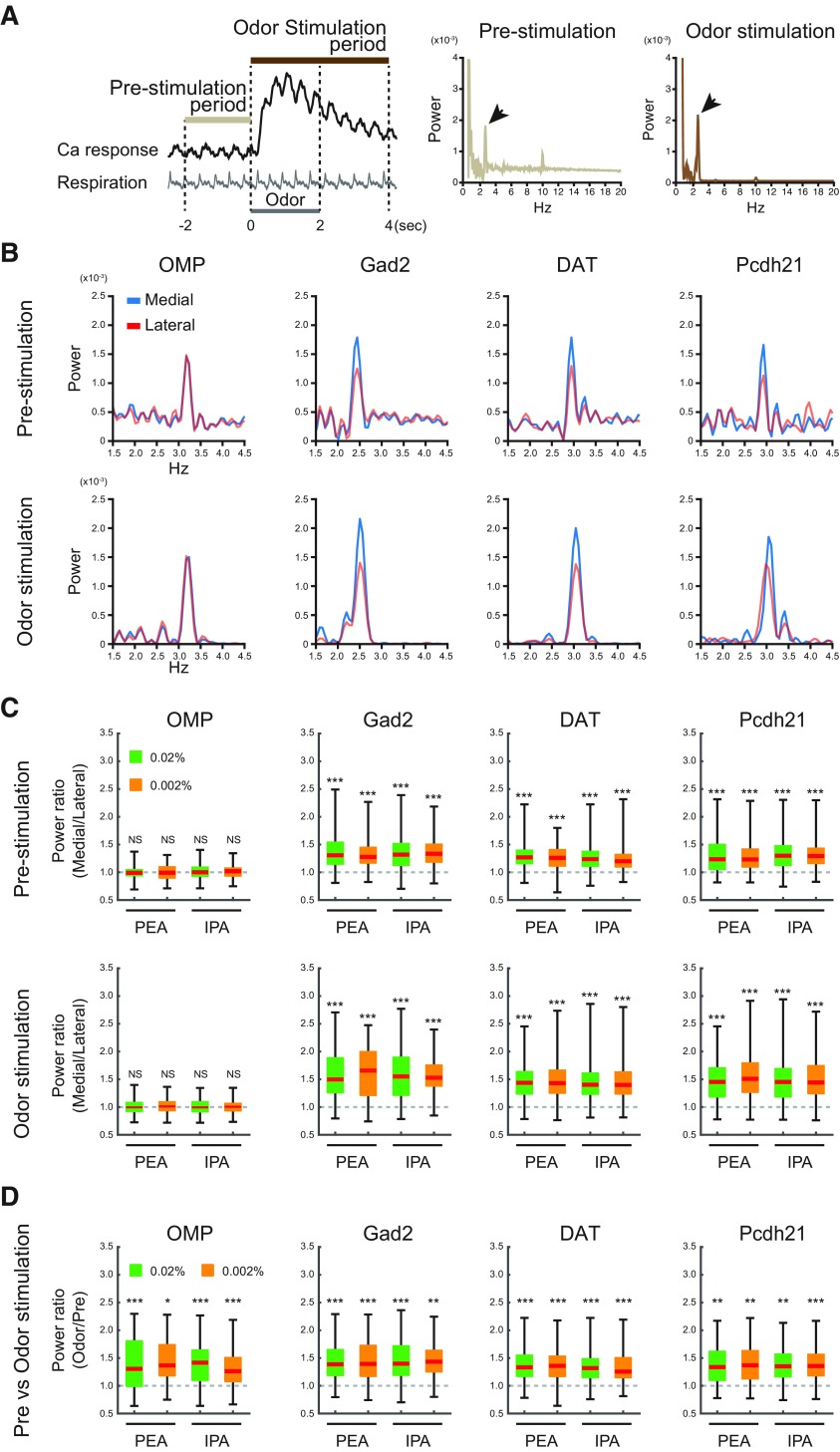
Respiration-locked calcium fluctuations of medial and lateral glomeruli. ***A***, Schematic illustration of data collection time points and power spectral analysis. The prestimulation and odor stimulation periods are defined in left panel. Middle and right panels are representative examples of power spectral analyses with large frequency ranges in single trials. Respiration-locked fluctuations of calcium signals were prominently observed at 2–4 Hz (arrows). ***B***, Representative results of power spectral analyses of calcium signals during prestimulation (upper rows) and odor stimulation (lower rows) periods in neurons from GCaMP-expressing OMP-Cre, Gad2-Cre, DAT-Cre, and Pcdh21-Cre mice. The calcium response traces of these spectra are shown as 0.02% IPA response signals in Fig. 2*B*. Blue and red traces indicate medial and lateral glomeruli, respectively (see also Extended Data Fig. 7-1). ***C***, Comparisons of power spectra between the homologous glomeruli during prestimulation (upper rows) and odor stimulation (lower rows) period in neurons from OMP-Cre, Gad2-Cre, DAT-Cre, and Pcdh21-Cre mice. The ratios of peak powers were calculated by dividing the medial glomerular power by the lateral glomerular power in each trial. The powers were calculated after correction for the white-noise background (see Materials and Methods). ***D***, Comparisons of peak power between the prestimulation and odor stimulation periods of the same glomeruli in each cell type. The power ratios were calculated by dividing the power value in the odor stimulation period by that in the prestimulation period after the subtraction of background level (see text); *p < 0.05, **p < 0.01, ***p <* *0.001 two-tailed paired Student’s *t* test.

Oscillatory calcium responses associated with respiration were observed in the optical recordings (Fig. 2*B*). These respiratory-linked fluctuations in calcium signals in medial glomeruli appeared to be larger in the postsynaptic neurons (i.e., GABAergic, dopaminergic, and mitral/tufted cells in the Gad2-Cre, DAT-Cre, and Pcdh21-Cre lines, respectively) than in the OSNs (i.e., cells in the OMP-Cre line). To examine this quantitatively, we applied a power spectral analysis to the data. Power spectra before and during odor stimulation displayed peak frequencies of 2–4 Hz (Fig. 7*A*, arrows), which matches the respiration rhythm under our experimental conditions. These respiration-locked calcium oscillations were detected in all the cell types (Fig. 7*B*). The peak power spectra at 2–4 Hz were indeed larger in medial glomeruli than in lateral glomeruli from Gad2-Cre, DAT-Cre, and Pcdh21-Cre mice both before and after odor stimulation but not in OMP-Cre mice. Because the power spectra background levels, which probably correspond to white noise, were higher in the prestimulation period than in the odor stimulation period (Fig. 7*A*), we analyzed the data after subtracting the background levels from the power spectra (Extended Data Fig. 7-1; see Materials and Methods); However, this did not alter the conclusions. The statistical analysis is summarized in Figure 7*C* (two-tailed paired *t* tests, see Table 2; the datasets are the same as in Figs. 3-6). These differences were observed consistently with 0.02% and 0.002% PEA and IPA (two-tailed paired *t* tests; Table 2). These results suggest that respiration-locked calcium fluctuations in the medial maps are enhanced postsynaptically in the OB. At last, as previous studies reported that the presence of odor stimulation enhances the oscillatory glomerular activity linked to respiration (or sniff; Spors and Grinvald, 2002; Carey et al., 2009), we compared the power between preodor stimulation period and odor stimulation period in the individual glomeruli (Fig. 7*D*). Consistent to the previous studies, larger respiration-locked modulation was observed during odor stimulation in all cell type, odorants, and concentrations we examined.

10.1523/ENEURO.0449-19.2020.f7-1 Extended Data Figure 7-1 Corrections for white-noise background in the power spectra. Representative power spectra of calcium signals from GCaMP-expressing OMP-Cre (***A1***), Gad2-Cre (***A2***), DAT-Cre (***A3***), and Pcdh21-Cre (***A4***) mice are presented. In each subpanel, the left and right columns represent the power spectra before and after correction for the white-noise background, respectively. The spectra in the upper and lower rows in the sub-panel are those from the prestimulation and the odor stimulation periods, respectively. The original plots in each mice (left columns in subpanels) are the same as those shown in Figure 7*B*. The background baseline was defined for each power spectrum as a mean value between 6 and 9 Hz. Then, these background values were subtracted from the original spectra. Download Figure 7-1, EPS file.

## Discussion

### Odorant response properties of homologous TAAR glomeruli in anesthetized animals

TAARs are a unique class of olfactory receptor; TAAR-expressing neurons project to the posteromedial glomeruli in the dorsal OB and are sensitive to predator odors (Liberles and Buck, 2006; Ferrero et al., 2011; Pacifico et al., 2012; Dewan et al., 2013; Zhang et al., 2013; Liberles, 2015). Taking advantage of the unique location of TAAR glomeruli, we compared, for the first time, the odorant response properties of homologous glomeruli in medial and lateral maps. While we did not observe clear differences in response amplitude, rising time, onset latency, or decay time between homologous glomeruli, the medial-map glomeruli showed larger respiration-locked fluctuations than those of lateral-map glomeruli, but only in postsynaptic cell types. One potential caveat in using TAAR glomeruli as a model is that the results may only be pertinent to TAAR glomeruli and may not reflect the properties shared across glomeruli regardless of the class of olfactory receptor. Therefore, it will be necessary to examine odorant response properties in a broader set of glomeruli to generalize the findings of this study.

In addition, it is also important to note that this study was conducted using anesthetized animals. Odor response properties are known to differ between anesthetized and nonanesthetized animals (Kato et al., 2012; Blauvelt et al., 2013; Ogg et al., 2018), probably because strong top-down modulation occurs in awake animals. In this study, we used anesthetized animals because the reduced efferent activity in these animals is an advantage in the initial characterization of afferent pathways. Future studies in nonanesthetized animals would help determine whether top-down inputs differentially modulate medial-map and lateral-map glomeruli, which could show that their activities are more diverse than those observed in this study, and would ultimately allow the activities of homologous glomeruli to be correlated with animal behavior.

### Presynaptic/postsynaptic calcium events in multiple cell types

In this work, GCaMP was expressed in postsynaptic GABAergic, dopaminergic, and mitral/tufted cells and in presynaptic axon terminals of OSNs of mice from various transgenic Cre-driver lines. Calcium signaling in OSNs reflects activation resulting in transmitter release, whereas calcium signaling in the other cell types may reflect activation of calcium-permeable glutamate receptors and the opening of calcium channels in response to excitatory postsynaptic potentials or spikes initiated in the dendrites or soma (Burnashev et al., 1992; Chen et al., 1997; Helmchen et al., 1999; Svoboda et al., 1999; Halabisky et al., 2000; Nagayama et al., 2007). Therefore, the calcium influxes in these cells are controlled by different biophysical mechanisms and represent different aspects of biological events. These differences would not impact our imaging results, as the comparisons were between medial and lateral glomeruli comprising the same cell types. Notably, we did not observe cell-type-specific differences between the medial and lateral maps at the glomerular level, which is consistent with recent data at the single-cell level showing the odorant responses of juxtaglomerular cells that are putatively associated with the same glomerulus (Homma et al., 2019). Nevertheless, we cannot exclude the possibility of differential response timing by different cell types, because the time resolution for calcium imaging was limited and did not reflect the activity of all neurons within a glomerulus. Future studies may begin to address this by imaging spike activity at a single-cell resolution.

### Response timing of the homologous glomeruli in medial and lateral maps

A pioneer electrophysiological study using a unique transgenic mouse line in which all OSNs express the same odorant receptor suggested that the latencies of mitral cell responses to odorant stimulation are shorter in the medial map than in the lateral map, especially at a low odorant concentration (Zhou and Belluscio, 2012). Because the OE is a complicated structure, airflow speed likely varies throughout the nasal cavity, and odorants may reach different areas at different times (Kimbell et al., 1997; Schoenfeld and Cleland, 2006; Zhao et al., 2006). This would produce a time lag for odorant responses in medial and lateral neurons. However, we did not observe different onset latencies between homologous medial and lateral glomeruli, even at low odorant concentrations. This may be because our recordings were via optical imaging rather than electrophysiology and from glomerular rather than MCLs. Another possible reason is the difference in OB regions recorded, which reflect inputs from different OE regions. Our study was restricted to a small region of the posteromedial dorsal OB, which receives inputs from the dorsal OE (Miyamichi et al., 2005). The dorsal OE faces the large nasal cavity and has a relatively simple wall structure compared with that of ventral zones. Moreover, OSNs projecting to medial and lateral maps are close together in the dorsal OE, for which any latency would not be detectable under our experimental conditions. It is possible that differential latencies from neurons in the ventral OB may be larger or more easily detected (Kimbell et al., 1997; Schoenfeld and Cleland, 2006; Zhao et al., 2006). In other words, the time lag may gradually become larger along the dorsal-ventral axis in the OB.

### Neuronal/circuitry mechanism of respiration-locked calcium fluctuations

The fluctuations of calcium responses, which corresponded to the rhythm of respiration, were larger in medial glomeruli than in lateral glomeruli. This may reflect differential airflow volumes or rates along the medial and lateral sides of the nostril. As this was only observed in postsynaptic calcium responses, the modulation is likely not within the OE but in the OB. The mechanism for this modulation may involve the physiological properties of these and/or associated neurons in medial and lateral glomeruli, such as differences in the expression of various calcium and/or other essential channels. As we did not observe differences in the amplitudes of the calcium responses, more than one channel type may be involved. Differential expression of other essential molecules would also change neuronal and/or network excitability. Neurons in medial glomeruli may also increase and decrease their intracellular calcium levels more synchronously during inhalation and exhalation, respectively. Such synchrony would be expected to affect the overall activity of neurons within a glomerulus; this could more directly be addressed by studies recording neuronal spikes in the context of a circuit. Thus, further investigations are needed to determine the mechanism by which calcium fluctuations are larger in medial glomeruli than in lateral glomeruli in response to odorant stimulation.

### Inhibitory connections between the two maps

Tufted cells in the lateral glomeruli project to cells in the internal plexiform and superficial GCLs underlying the medial glomeruli receiving inputs from the same odorant receptors, and vice versa. These projections activate granule cells and thus inhibit surrounding mitral/tufted cells (Belluscio and Katz, 2001; Lodovichi et al., 2003; Zhou and Belluscio, 2008), resulting in mutual inhibition between the medial and lateral maps. Our data suggest this inhibition is not simple (i.e., one glomerulus is inhibited when the other is activated), as medial and lateral glomeruli are activated simultaneously during odor stimulation. The similar temporal patterns and the absence of counterphase-locked activity between the two glomeruli also suggest that the activity of one glomerulus does not inhibit the other homologous glomerulus in a given time phase, such as during inhalation or exhalation. The functional role of these mutual inhibitory connections and how they contribute to odor processing remain unknown. It is possible that these connections regulate the temporal activity pattern and/or synchrony of neurons or glomeruli in both maps.

### Odor information processing streams from the medial and lateral maps

One of the unresolved issues is where the medial and lateral maps project and how higher brain centers handle these two outputs. Current knowledge of the connections between the OB and the olfactory cortex (Nagayama et al., 2010; Ghosh et al., 2011; Miyamichi et al., 2011; Sosulski et al., 2011; Igarashi et al., 2012) suggest that there may not be dramatic differences regarding where the maps project. However, it is still not known whether the outputs from the two maps are evenly transmitted to all olfactory cortical areas, and some regions may preferentially receive input from one or the other. More detailed knowledge of the cortical projections may help reveal the significance of the multiple maps and how the information from the two information streams is compiled in higher brain centers.

### Rhythm of respiration

In vertebrate land animals, airflow through the nasal cavity during respiration alternates between orthonasal and retronasal directions. This induces a synchronized rhythm in the olfactory system, which contributes to the odor information process (Spors and Grinvald, 2002; Wilson and Mainen, 2006; Cury and Uchida, 2010; Uchida et al., 2014). Moreover, the orthonasal and retronasal airflows switch the perception from smells originating from the surrounding environment to taste in the mouth, respectively (Gautam and Verhagen, 2012; Shepherd, 2012). In addition to olfactory areas, respiration-locked oscillations have been observed in hippocampus as well as barrel and prefrontal cortices in the rodent (Shusterman et al., 2011; Phillips et al., 2012; Yanovsky et al., 2014; Lockmann et al., 2016; Nguyen Chi et al., 2016; Biskamp et al., 2017). In the barrel cortex, phase-locked oscillation patterns coordinate the interaction between olfaction and tactile sensations (Ito et al., 2014), whereas freezing behavior is modulated by rhythmic activity in prelimbic prefrontal cortex driven by inputs from the anterior olfactory nucleus (Moberly et al., 2018), which has topographical connections to the OB (Schoenfeld et al., 1985; Yan et al., 2008). The anterior olfactory nucleus sub-region, which dominantly receives inputs from the medial map, may relay this information to higher brain centers controlling respiration-linked neuronal activity associated with mouse behavior. Thus, the anterior olfactory nucleus may be responsible for processing information from multiple glomerular maps and represents an area that warrants further study.

## Synthesis

Reviewing Editor: Anna Menini, SISSA (International School of Advances Studies) Decisions are customarily a result of the Reviewing Editor and the peer reviewers coming together and discussing their recommendations until a consensus is reached. When revisions are invited, a fact-based synthesis statement explaining their decision and outlining what is needed to prepare a revision will be listed below. The following reviewer(s) agreed to reveal their identity: Veronica Egger, Dinu Florin Albeanu. Note: If this manuscript was transferred from JNeurosci and a decision was made to accept the manuscript without peer review, a brief statement to this effect will instead be what is listed below.

In the olfactory bulb there are mostly two target glomeruli for each type of OR and thus two parallel representations of receptor channels. In this study, the authors tackle the long-standing and up to now strikingly understudied question on the purpose of this mirror glomeruli system by investigating potential differences in signal processing. Since usually the two glomeruli are not (yet) simultaneously accessible to optical recordings from one imaging plane because of their anatomical arrangement, here the authors make use of the fortuitous fact that within the representation of the TAAR family, both glomeruli are located within the dorsal surface of the bulb and thus their responses can be detected in parallel.

The study relies on four different transgenic mouse lines that express GECI either in the OSNs (presynaptic label) or in two different subtypes of glomerular inhibitory neurons (periglomerular/DAergic cells) or in principal neurons (apical tufts of mitral/tufted cells) and uses Ca2+ imaging in vivo (anesthetized) of responses to two amines at two concentrations. The authors present convincing evidence that the signals in lateral vs medial glomeruli are highly similar, with the exception of the power of the respiration-coupled oscillatory component of the Ca2+ signals. For all mouse lines except for the presynaptic label this oscillatory power is higher for the medial glomeruli both at rest and during odor presentation, when the difference is further increased. Potential mechanisms for this difference are discussed.

This is a first report providing information on the response dynamics of mirror image TAAR glomeruli in anesthetized mice. The use of anesthesia might actually correspond to a reductionist approach that studies a primary, unmodulated response of the bulb. This study is a very interesting starting point for understanding potential differences in the responses of mirror glomeruli in awake and behaving mice. Indeed, in awake behaving animals, engaged in olfactory behaviors, potential differences might become apparent, as various stimulus configurations, different odors and concentrations and additional glomeruli are probed.

The revised manuscript is much improved but some minor points still need to be addressed. Please respond to the following comments and make appropriate revisions to your manuscript.

Reviewer #1

The authors have addressed most of my queries in a satisfactory manner. In particular, they now discuss the two main issues that might preclude a generalization of the results to olfactory bulb processing across any pair of medial/lateral glomeruli - anesthesia and potentially specific processing by the TAAR-associated glomeruli.

They do so very prominently in the first paragraph of the discussion, and I also value their argument that use of anesthesia might actually correspond to a reductionist approach that studies a primary, unmodulated response of the bulb.

However - or rather, all the more - I think it would be appropriate to mention the use of anesthesia in the abstract! I also asked the authors in the previous review to mention the TAARs in their conclusion at the end of the abstract (l.54 f) which they neglected to do. I think they should at least tone down their generalizing statement a bit.

E.g. This indicates that the medial rather than the lateral OB map MIGHT enhance the respiration-locked rhythm and transfer this information to higher brain centers.

It also seems to me that my last minor comment on Fig 8 (former numbering) was perhaps misunderstood. I do not understand why the authors refuse to acknowledge that there is more power in the oscillation in the presence of odors (at least as obvious from their examples in 8A and B and that this observation has been made earlier (e.g. here, “With odor stimulation, the amplitude of the respiration-synchronous modulation increases.” (citation from Spors et al 2002 VSD imaging, their Fig 6) or in particular Fig 1 in Carey Wachowiak 2009 which also uses presynaptic Ca2+ imaging). Interestingly these older data do not show pre-odor oscillations for all glomeruli, whereas such oscillations seem to always have been detected in the study under review. Is this a technical issue (better SNR) or something systematic that the TAAR glomeruli always showed but not others?

Actually, former Fig 8 D is a control for their experimental paradigm if the data was properly reanalysed (with substraction of white noise) and as such could go into a supplement or into the control figure 1.
